# TRIDENT cognitive substudy: preliminary results from a Brazilian cohort

**DOI:** 10.1002/alz.088874

**Published:** 2025-01-03

**Authors:** Ana Cláudia de Souza, Wyllians Vendramini Borelli, Kendi Nishino Miyamoto, Ana Claudia Alves, Mariana Almudi, Adriana Binsfeld Hess, Renata Kochhann, Leonardo Augusto Carbonera, Sheila O Martins, Craig S. Anderson

**Affiliations:** ^1^ Hospital Moinhos de Vento, Porto Alegre, Rio Grande do Sul Brazil; ^2^ Universidade Federal do Rio Grande do Sul, Porto Alegre, Rio Grande do Sul Brazil; ^3^ Hospital Moinhos de Vento, Brazil, Rio Grande do Sul Brazil; ^4^ Federal University of Rio Grande do Sul (UFRGS), Porto Alegre Brazil; ^5^ The George Institute for Global Health, Newtown, NSW Australia

## Abstract

**Background:**

Intracerebral hemorrhage (ICH) is the most severe and disabling stroke, accounting for up to 50% of the cases in low‐to‐middle‐income countries. High rates of cognitive decline and dementia follow acute ICH, due to the common underlying vasculopathy of cerebral small vessel disease (CSVD). The international clinical trial, TRIDENT (Triple therapy prevention of Recurrent Intracerebral Disease EveNts Trial), aims to determine the effectiveness of the fixed low‐dose Triple Pill combination of blood pressure‐lowering agents (telmisartan 20 mg, indapamide 1.25 mg, and amlodipine 2.5 mg) versus placebo on recurrent stroke and additionally on the secondary outcomes of cognitive decline and dementia. The aim of the Brazilian substudy is to assess the Triple Pill effectiveness on memory, cognition, and CSVD progression in ICH patients.

**Method:**

This substudy is part of the TRIDENT international, multicenter, double‐blind, placebo‐controlled, parallel‐group, randomized controlled trial led by The George Institute for Global Health in Australia. A subset of participants recruited from Brazilian local sites are offered the opportunity to undergo additional cognitive assessments through gold‐standard neuropsychological tests at baseline (until up to 6 weeks post‐randomization), and 12 months. MRI evaluation of these patients will be performed to assess CSVD progression through specific parameters at baseline and 12 months.

**Primary Outcome (MRI) ‐** Changes in T2 FLAIR WMH volume between baseline and 12 months will be assessed.

**Secondary Outcomes (MRI)**

1. Whole brain atrophy measured by percentage brain volume change between baseline and 12 months on HIRES‐T1.

2. Substructure change (cortical grey matter, white matter, and CSF) between baseline and 12 months.

3. Change in the number of CMBs between baseline and 12 months on SWI.

**Result:**

The study recruitment is ongoing.

**Conclusion:**

The substudy could potentially evaluate Triple Pill’s effectiveness on cognitive decline, dementia, and CSVC progression.

